# Dendritic Cell-Based and Other Vaccination Strategies for Pediatric Cancer

**DOI:** 10.3390/cancers11091396

**Published:** 2019-09-19

**Authors:** Sévérine de Bruijn, Sébastien Anguille, Joris Verlooy, Evelien L. Smits, Viggo F. van Tendeloo, Maxime de Laere, Koenraad Norga, Zwi N. Berneman, Eva Lion

**Affiliations:** 1 Division of Hematology, Antwerp University Hospital, Wilrijkstraat 10, 2650 Edegem, Antwerp, Belgium; severine.debruijn@student.uantwerpen.be (S.d.B.); zwi.berneman@uza.be (Z.N.B.); 2 Center for Cell Therapy & Regenerative Medicine, Antwerp University Hospital, Wilrijkstraat 10, 2650 Edegem, Antwerp, Belgium; evelien.smits@uza.be (E.L.S.); maxime.delaere@uza.be (M.d.L.); eva.lion@uantwerp.be (E.L.); 3 Tumor Immunology Group, Laboratory of Experimental Hematology, Vaccine & Infectious Disease Institute (VAXINFECTIO), Faculty of Medicine & Health Sciences, University of Antwerp, 2610 Wilrijk, Antwerp, Belgium; viggovantendeloo@gmail.com; 4 Division of Pediatric Hemato-Oncology, Antwerp University Hospital, Wilrijkstraat 10, 2650 Edegem, Antwerp, Belgium; joris.verlooy@uza.be (J.V.); koen.norga@uza.be (K.N.); 5 Center for Oncological Research, Faculty of Medicine & Health Sciences, University of Antwerp, Universiteitsplein 1, 2610 Wilrijk, Antwerp, Belgium

**Keywords:** dendritic cells, pediatric cancer, tumor vaccination, immunotherapy

## Abstract

Dendritic cell-based and other vaccination strategies that use the patient’s own immune system for the treatment of cancer are gaining momentum. Most studies of therapeutic cancer vaccination have been performed in adults. However, since cancer is one of the leading causes of death among children past infancy in the Western world, the hope is that this form of active specific immunotherapy can play an important role in the pediatric population as well. Since children have more vigorous and adaptable immune systems than adults, therapeutic cancer vaccines are expected to have a better chance of creating protective immunity and preventing cancer recurrence in pediatric patients. Moreover, in contrast to conventional cancer treatments such as chemotherapy, therapeutic cancer vaccines are designed to specifically target tumor cells and not healthy cells or tissues. This reduces the likelihood of side effects, which is an important asset in this vulnerable patient population. In this review, we present an overview of the different therapeutic cancer vaccines that have been studied in the pediatric population, with a main focus on dendritic cell-based strategies. In addition, new approaches that are currently being investigated in clinical trials are discussed to provide guidance for further improvement and optimization of pediatric cancer vaccines.

## 1. Introduction

In the Western world, cancer is the second most common cause of death among children aged 1 to 14 years, surpassed only by traumatic injuries [1,2]. Approximately 11,000 children (0–14 years) will be diagnosed with cancer in 2019, most frequently with leukemias, or brain and other nervous system tumors. Advances in conventional treatment modalities (i.e., chemotherapy, radiation, surgery and hematopoietic stem cell transplantation [HSCT]) have improved the 5-year relative survival rate for childhood cancers from 58% during the mid-1970s to approximately 83% [3]. The prognosis for children with refractory and relapsed malignancies is, however, associated with dismal outcomes, regardless of therapy intensification [4]. Furthermore, due to long-term toxicity from the intense chemotherapy and radiation regimens, those who do recover are at elevated risk of health consequences later in life, including a higher risk for developing secondary cancers [5]. More targeted therapies are required to overcome these treatment-related side effects and to further improve survival with a favorable long-term quality of life [4,6].

**Figure 1:**
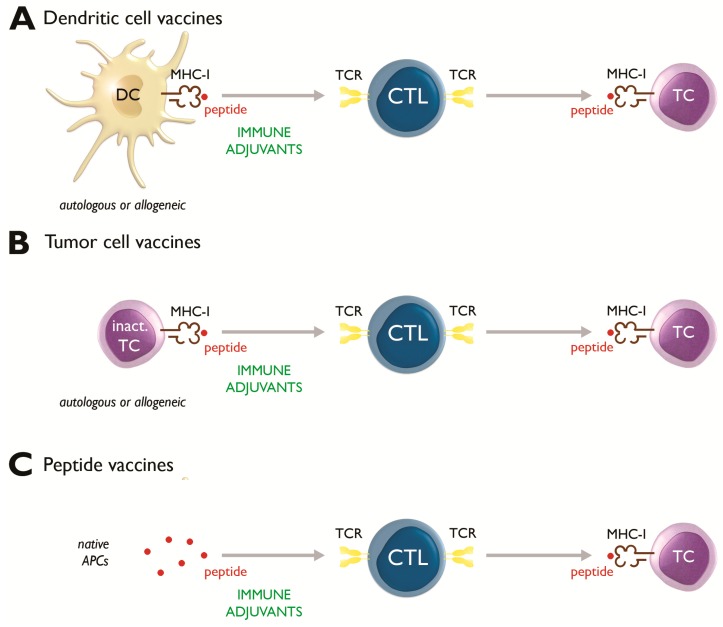
Mechanistic principles of anti-cancer vaccination. The most important types of tumor vaccines are dendritic cell (DC), tumor cell (TC), and peptide vaccines. The common mechanism of action for all tumor vaccines is the induction of tumor antigen-specific cytotoxic T-lymphocytes (CTLs). These CTLs are capable of recognizing and killing TCs that express tumor antigen fragments, designated peptides, on their cell surface in the context of major histocompatibility complex (MHC) molecules. The recognition of the peptide/MHC is conferred by the T-cell receptor (TCR). (**A**) DCs are professional antigen-presenting cells (APCs) and are thus highly equipped to induce tumor antigen-specific CTLs. DCs, either from autologous or allogeneic origin, can be loaded with antigenic material through different ways (e.g., by pulsing with peptides, or with TC lysates). These tumor-antigen loaded DCs are usually administered in combination with immune adjuvants for improved immune stimulation. (**B**) Autologous or allogeneic TCs, inactivated (inact.) by lysis, can also be used in combination with immune adjuvants to induce tumor antigen-specific CTLs, which are in turn capable of killing TCs that express the corresponding tumor antigenic peptide(s). (**C**) Peptides, administered together with immune adjuvants, are also being used for therapeutic cancer vaccination purposes. Peptide vaccine-based approaches rely on the presence of functionally competent APCs in vivo for effective stimulation of a CTL immune response.

For at least two centuries, attempts have been made to use the immune system to fight cancer [7]. A broad spectrum of anticancer immunotherapeutic agents are undergoing intensive investigation, including passive (e.g., tumor-targeting monoclonal antibodies, oncolytic viruses, and adoptively transferred T cells) and active (e.g., therapeutic cancer vaccines, immunostimulatory cytokines, and checkpoint inhibitors) forms of immunotherapy [8,9,10,11,12]. Instead of preventing cancer, therapeutic tumor vaccines are designed to treat existing cancers, mainly by stimulating a cytotoxic T-lymphocyte (CTL) immune response against particular antigens expressed on the surface of tumor cells in the context of major histocompatibility complexes (MHC) [11,13]. Tumor antigen-loaded dendritic cells (DCs) have attracted much attention as cancer vaccine vehicles [8,14]. Other cancer vaccination strategies include the use of whole tumor cells and peptides (Figure 1). As tumor vaccines mobilize several immune effector mechanisms that precisely attack and destroy cancer cells, sparing normal cells, they are attractive treatments for advanced and/or relapsed pediatric cancers. Yet most therapeutic cancer vaccine trials have been performed in adult populations; children are an understudied population [15,16,17]. Nevertheless, results from clinical trials in adults cannot be simply extrapolated to children, even if the histology of the targeted tumor is the same. Within a specific histological tumor type, the type and frequency of molecular/genetic abnormalities can differ significantly between adults and children [18]. Furthermore, the treatment success is generally better in the pediatric age groups, most likely due to better treatment tolerance in children and also due to a “simpler” tumor biology that may be more amenable to treatment. In the case of immunotherapy, there are specific considerations of safety in the pediatric age group that relate to the immaturity of the immune system. Additionally, the pediatric immune system is more adaptable and vigorous than that of an adult, and immunocompetence can be reconstituted in children even after multiple cytotoxic therapies [6,19]. As a consequence, the response to immunotherapy can differ between children and adults. This underscores the need to undertake separate clinical studies to establish the safety and efficacy of tumor vaccines, and by extension other immunotherapies, in children. In this review, we will discuss the progress and limitations of different types of tumor vaccines, including DCs, whole tumor cells and peptide vaccines, that are currently being investigated in pediatric cancer patients.

## 2. Therapeutic Vaccination for Pediatric Cancer: The Past and the Present

In recent years, a better understanding of tumor biology and its interaction with the immune system, along with improved strategies for vaccine development, have led to the development of different potential vaccines against specific cancers [4,20]. Therapeutic cancer vaccines can be categorized as cellular vaccines (consisting of DCs or autologous/allogeneic tumor cells), protein/peptide vaccines, and genetic (DNA, RNA and viral) vaccines. Each type of vaccine has its specific advantages and disadvantages [19]. In the pediatric population, most clinical trials have relied on DCs as cellular tools to stimulate a tumor antigen-specific CTL immune response (Table 1, Figure 1a), although tumor cell preparations (Table 2, Figure 1b) and peptide vaccines (Table 3, Figure 1c) have also been used. To the best of our knowledge, no studies have been published with genetic vaccines in pediatric cancer patients.

As shown in Table 1, Table 2 and Table 3, cancer vaccines have been applied in different disease types: Pediatric brain cancer, a variety of other solid tumors (e.g., sarcoma and neuroblastoma), as well as hematological malignancies (e.g., acute lymphoblastic leukemia [ALL] and acute myeloid leukemia [AML]) (Table 1, Table 2 and Table 3). Brain tumors, especially gliomas, have been the most extensively studied tumor type. Most gliomas can be categorized as either low-grade (LGGs) or high-grade gliomas (HGGs). The latter includes anaplastic astrocytoma (AA) and glioblastoma multiforme (GBM) [6,21,22].

In general, therapeutic cancer vaccination was usually applied in a relapsed/refractory setting, after conventional treatments. The study populations were small due to the rareness of the different cancer types and the early-phase design of most studies. To the best of our knowledge, only one phase III, randomized, double-blind, placebo-controlled clinical study has been undertaken (Clinical trials.gov identifier NCT03520959), but this study was terminated prematurely and no results were released. All other studies were of phase I or II design and none were randomized. This exemplifies that the traditional phased approach in clinical research is extremely challenging in the pediatric population. The rarity of childhood cancers may offer a partial explanation for this, because it often precludes the initiation of large phase II or III studies. In addition, most clinical trials are first being undertaken in adults; it is ethically difficult to repeat randomized studies in children, especially if the drug or treatment has already shown signs of clinical activity in adults. Of note, the age range varied considerably between the different studies listed in Table 1, Table 2 and Table 3, with some studies also including (young) adults (Table 1, Table 2 and Table 3).

### 2.1. Dendritic Cell-Based Vaccines

Table 1 lists all DC vaccination trials that have been performed so far in pediatric cancer patients. DCs are specialized antigen-presenting cells (APCs), and can capture, process and present pathogen- or host-derived antigenic peptides, in the context of both MHC class I and II molecules, to naïve CD4^+^ or CD8^+^ T-lymphocytes, respectively [19,23]. Additionally, DCs can initiate, maintain and regulate the intensity of primary immune responses, including protective antitumor responses. Besides their important role in the adaptive immune response, DCs are also strong activators of natural killer (NK) cells, effectively linking innate and adaptive immune responses [7,24,25].

A possible limitation to the wide use of DCs as cancer vaccines is the process required to generate the vaccine. To make DC vaccines, a large number of peripheral blood mononuclear cells must first be collected, usually via leukapheresis. A leukapheresis procedure typically yields 3–16 × 10^9^ peripheral blood mononuclear cells [26,27,28,29]. These cells can be obtained from the patient (autologous) or, in the event that the patient has undergone an allogeneic HSCT, from the donor (allogeneic). Cells are then cultured with cytokines, typically granulocyte-macrophage colony-stimulating factor (GM-CSF) and interleukin (IL)-4, and loaded with antigenic material, which can sometimes be obtained by invasive procedures only (this is, for example, the case if autologous tumor cell preparations are used to load the DCs with) [30]. Furthermore, adjuvants are usually required to stimulate a vigorous immune response, especially if weakly immunogenic tumor-related antigens are used [4]. Toll-like receptor (TLR) ligands, such as Imiquimod, and Keyhole-limpet hemocyanin (KLH) are examples of appropriate immune adjuvants [31]. Cytokines (e.g., IL-2 or IL-7) are also often used as immune stimulants (Table 1). These adjuvants are usually administered to the patients concomitantly with the DCs. DC vaccine administration can be performed through the intradermal (ID), intravenous (IV), subcutaneous (SC), or intranodal (IN) routes (Table 1). The majority of the studies listed in Table 1 employed ID vaccine injection. Theoretically, optimal T-cell activation would be expected from intradermally administered antigen-pulsed DCs migrating to the regional draining lymph nodes where maximal contact with T cells can be established [32,33,34].

Since a universal tumor-specific antigen has not (yet) been identified and due to the inter-individual heterogeneity in tumor antigen expression, most studies have used tumor material (cell lysates or whole tumor RNA) as a source for loading the DCs with antigens. This offers the advantage that the entire repertoire of tumor-related antigens can be presented by the DCs. Of the trials performed with tumor lysate-pulsed DCs, only one study employed allogeneic tumor cell lines in children with newly diagnosed diffuse intrinsic pontine glioma (DIPG, WHO grade II-IV gliomas) [35]. All other studies used autologous tumor cell material. One study group used autologous DCs pulsed with whole tumor RNA in children and young adults with recurrent brain tumors [26]. Alternatively, DCs can also be loaded with antigen by peptide pulsing. The group of Mackall et al. has used peptides derived from tumor-specific translocation breakpoints in patients with translocation-positive sarcomas [36,37]. Other peptides used include a combination of cancer-testis antigens (MAGE-A1, MAGE-A3, NY-ESO-1) [38], and peptides derived from the Wilms’ tumor 1 (WT1) protein [39,40]. *WT1* was first identified as an oncogene involved in the development of Wilms’ tumor, a childhood renal cancer [41]. Nevertheless, WT1 has been identified as a target antigen of other solid tumors and of hematological malignancies [41,42], such as AML where it is expressed at high level [43,44]. Clinical trials of WT1-targeted DC vaccination in adult patients with AML have already shown promising results [14,45].

In line with what has been observed in adults [8], all studies listed in Table 1 confirmed the feasibility of generating DC vaccines in pediatric patients. The treatment was also well tolerated, with local injection site reactions being the most commonly reported adverse events. Systemic toxicity, if any, was generally mild; only two patients with gliomas experienced a grade IV toxicity, which could be effectively treated with corticosteroids [28,46]. In the study by Merchant et al., one patient had a grade IV fever and grade IV anaphylaxis, but this was attributed to recombinant human (rh) IL-7 that was co-administered [47]. No other major toxicities were reported, confirming that DC vaccination is a safe treatment modality even in heavily pre-treated pediatric cancer patients.

The immunological principle of DC vaccination is the induction of a vigorous antigen-specific T-cell (adaptive) immune response. As shown in Table 1, tumor antigen-specific T-cell immune responses have been observed in various studies. The group of Benitez-Ribas et al. has shown that antigen-reactive T cells can be induced not only in the periphery but also in the cerebrospinal fluid in children with DIPG [35]. Dohnal et al. observed increased tumor antigen-specific T-cell reactivity only after IN DC vaccination, but not after SC administration [48]. This indicates that the SC administration route may be suboptimal, which is in line with the general assumption that subcutaneously administered DCs are less immunogenic because of their reduced ability to migrate toward the lymph nodes [49]. In addition to increased T-cell reactivity, numerous studies have also reported delayed-type hypersensitivity (DTH) reactions, indicating induction of cellular immunity by the DC vaccination [50,51]. Other immunological phenomena observed after DC vaccination in pediatric patients include: induction of humoral immunity [26], increases in (activated) CD8^+^ T cells [52,53] and NK cell activation [52] (summarized in Table 1). The finding that DC vaccination induces NK cell responses is of particular interest given the increasingly recognized role of NK cells in DC-initiated antitumor immunity. Besides direct killing of tumor cells [54], NK cells are also capable of amplifying DC-induced antitumor responses. For example, NK cells facilitate cross-presentation of tumor-derived antigens to CTLs [24,55]. Furthermore, bidirectional crosstalk between DCs and NK cells prompts enhanced activation of both cell types, thereby augmenting their antitumor potential. Alternatively, if not subject to NK-stimulated maturation, immature DCs are lysed by NK cells, preventing inappropriate tolerization of T cells [24,55]. Interestingly, in the study of Suminoe et al., enhanced NK cell cytotoxic activity was only observed in pediatric patients displaying a positive clinical response following DC vaccination [52]. This correlation, which has also been observed in several adult studies, underscores the physiological relevance of DC vaccine-associated NK cell responses in tumor control [7,8,24,55].

Due to their early-phase design, clinical outcomes were often a secondary objective in most of the hitherto performed clinical trials. Nevertheless, most studies have reported clinical outcome data (Table 1). In children with solid tumors, objective clinical responses according to the RECIST criteria have been sporadically documented, including complete (CR) and partial responses (PR), as well as mixed responses (MR). In addition, stable disease (SD) has been observed in a considerable number of patients (Table 1). It is important to note that DC vaccines were sometimes combined with other therapies, making it difficult to assess the single-agent clinical activity of DC vaccination. For example, in the study by Krishnadas et al. in which a CR was documented in one patient with neuroblastoma, MAGE-A1/MAGE-A3/NY-ESO-1 peptide-pulsed DCs were combined with the hypomethylating agent decitabine [38]. This combination is likely to produce synergistic effects, since decitabine facilitates epigenetic upregulation of the cancer-testis antigens MAGE-A1, MAGE-A3 and NY-ESO-1. In patients with hematological malignancies, conversion of minimal residual disease (MRD)-positive into MRD-negative remissions was demonstrated [53], although in that study DC vaccination was also combined with another treatment modality (i.e., cytokine-induced killer cells).

Overall, as becomes evident from Table 1, best clinical responses were observed in patients with limited disease or those with CR, where DC vaccination can maintain the CR state and prevent recurrence. DC vaccine therapy appears to be less effective in patients with (high) residual tumor load or progressive disease (PD), most likely because of the overwhelming immunosuppressive burden imposed by the cancer cells and because there is insufficient time to mount an anti-tumor immune response. The impact of residual tumor burden on clinical outcome was indeed demonstrated in several studies. Ardon et al. observed a better median progression-free survival (PFS) in the relapsed HGG group when a total resection of the tumor was achieved (8.8 months) versus subtotal resection (3 months), respectively [56]. In a study by Lasky et al., two of three patients, both with disease that could not be detected by imaging, experienced SD while the third patient with subtotally resected disease progressed [28]. Although Dohnal et al. and Mackall et al. used the same type of DC vaccine, the latter study group achieved better survival results. This can be explained by the fact that all patients in the study by Dohnal et al. had macroscopic disease at the start of vaccination compared to Mackall et al., where the vaccine was given as consolidative therapy during the period of clinical remission following multimodal therapy [37,48]. A similar observation was made in DC vaccine studies in adults, both in solid cancers [8] as well as in hematological malignancies [14].

Although it is challenging to assess survival benefit in single-arm studies, several of the clinical trials listed in Table 1 have reported unexpectedly long survival times. For example, De Vleeschouwer et al. noted that DC vaccine-treated patients under the age of 35 had an overall survival (OS) of 15.3 months, which was an improvement compared to a Children’s Cancer Group trial which reported that all patients with recurrent malignant glioma died within 1 year [6,46]. In patients with Ewing’s sarcoma, the combination of tumor lysate/KLH-pulsed DC vaccinations with autologous lymphocytes and rhIL-7 administered following standard antineoplastic therapy resulted in higher survival rates and lower recurrence rates compared with patients receiving standard antineoplastic therapy alone [47]. Merchant et al. achieved a 5-year intent-to-treat OS of 77% in patients with newly diagnosed metastatic Ewing’s sarcoma/rhabdomyosarcoma, which is higher than usually observed in this population [47]. Based on data from the same group, the expected 5-year OS rate in this population is 25% [37]. Although survival claims are difficult to prove in the absence of randomized trials, these observations hint at a possible OS advantage for DC vaccine-treated children with cancer, similar to what has been observed in adults [8].

### 2.2. Tumor Cell Vaccines

Autologous or allogeneic tumor cells accompanied by an immune stimulant, such as GM-CSF, can also serve as cancer vaccines (Figure 1b). One of the major advantages of whole tumor cell vaccines is their potential to present the entire spectrum of tumor antigens, both known and undefined, meaning that target antigens need not be prospectively identified [19,57]. However, this same quality can potentially reduce the relative level of expression of potentially relevant tumor antigens. Producing autologous tumor cell vaccines depends on the availability of tumor biopsies, limiting the feasibility of this approach to patients with accessible tumor sites [58]. Allogeneic vaccines, derived from tumor cell lines and irradiated to prevent further cell division, overcome the logistical limitations of autologous tumor cell vaccines and permit standardized and large-scale production, easy manipulation for expression of immunostimulatory molecules and cost-effectiveness [19].

As shown in Table 2, tumor cell vaccines, both autologous and allogeneic, have already been studied in children with neuroblastoma and hematological malignancies (Table 2). Neuroblastoma is the most common solid extra-cranial tumor in children, accounting for more than 7% of malignancies in patients younger than 15 years old and around 15% of all pediatric oncological deaths [59]. Prognosis for these patients is variable at different ages due to the heterogeneity of the tumor and its biological behavior, which make it difficult to predict the prognosis and course of neuroblastoma [60]. There are few defined antigens known to be consistently associated with neuroblastoma [61]. Whole tumor cell vaccines, composed of cellular extracts rather than individual proteins or peptides, are therefore an attractive choice for these types of tumor as such vaccines allow multiple tumor antigens to be presented. This overcomes the issue of antigen heterogeneity between and within neuroblastoma tumor samples [4].

Bowman et al. examined treatment with interleukin (IL)-2 gene-modified tumor cells, both allogeneic [62] and autologous [63], for the treatment of relapsed or advanced neuroblastoma. The autologous vaccines were found to be more immunostimulatory and showed a superior antitumor cytotoxic immune response. One of the ten patients treated with the autologous vaccine had complete tumor regression. The superiority of autologous over allogeneic whole tumor cell vaccines is most likely due to the fact that autologous tumor vaccines not only express shared antigens but also unique antigens specific to each individual tumor [6]. In the autologous trial, neuroblasts were transduced with an adenoviral vector encoding IL-2. The presence of adenoviral antigens on the surface of the transduced autologous cells may have produced an adjuvant-like effect in the majority of adenovirus-immune patients, ensuring a milieu favorable to recruitment and amplification of tumor-specific T lymphocytes [62,63]. The same autologous adenoviral-based vaccine was later tested in patients with high-risk neuroblastoma in remission [64]. Eight patients were in first remission and 5 in second or later remission. There was a major difference between first remission patients and patients who were treated for relapse, with a median PFS of 22 and 3 months, respectively. This again indicates that therapeutic cancer vaccines are more likely to be successful in the setting of MRD and in less pretreated patients [64]. Other trials, one autologous [65] and one allogeneic [66], tested an engineered vaccine expressing both IL-2 and lymphotactin in patients with advanced or refractory neuroblastoma. The synergistic effect of IL-2 in association with lymphotactin, a T cell-attracting chemokine, leads to systemic immunity capable of rejecting growing tumors in murine models [67]. In patients, SD was observed as the best clinical response.

Two groups attempted vaccination of patients with high-risk leukemia using autologous malignant blasts. Haining et al. treated nine patients (aged 5–60 years) with relapsed or refractory ALL using a vaccine composed of autologous malignant blasts stimulated with CD40 ligand. Only 2 of the nine included patients received the vaccine; most patients progressed or died before the vaccination could be initiated. This led the authors to conclude that autologous tumor vaccination was not feasible at the time of relapse, because aggressive leukemia progresses rapidly and there is insufficient time to harvest cells and construct the vaccine [68]. Rousseau et al. used irradiated autologous malignant blasts mixed with autologous fibroblasts transduced to express human CD40 ligand and human IL-2, in ten patients, including seven children with AML or ALL in cytologic remission. The vaccine was proven to be safe and anti-leukemia humoral and Th1 type immune responses were generated. Furthermore, the relapse-free survival was 85% at 3 years, which is higher than traditional controls [69].

### 2.3. Peptide Vaccines

Peptide vaccines are recombinant vaccines based on peptides derived from defined tumor-associated antigens. These peptides are usually administered with an adjuvant and/or with an immune modulator to augment antigen immunogenicity [58]. Compared to cellular vaccines which require cell collection and processing, peptide vaccines are generally cost-effective and easy to administer. Moreover, multi-antigen-specific immune responses can be acquired by administration of a peptide cocktail that contains different tumor antigens. However, the number of antigen targets for the immune response is ultimately restricted, which can result in possible immune escape due to positive clonal selection for antigen-loss variants. Thus, those tumor cell clones that do not express the particular tumor antigen or clones that undergo immune editing and lose expression of the target antigen will potentially escape immune rejection and, consequently, have significant proliferation advantages compared to cell clones that express the targeted tumor antigen [35].

Most published clinical trials of peptide vaccination in the pediatric population have used peptides derived from the WT1 antigen (Table 3). WT1 is a nearly universal tumor-associated antigen that is expressed in a broad range of solid and hematological malignancies. The frequency of WT1 overexpression in pediatric cancer patients appears to be lower than it is in adults. Despite this, a study that included both relapsed/refractory solid and hematological malignancies by Sawada et al. found that more than 50% of pediatric patients tested positive for WT1 expression, which indicates that the WT1 peptide vaccine is applicable to a large proportion of pediatric cancer patients [72]. Another study by Hirabayashi et al., who investigated WT1 peptide vaccination in a mixed group of pediatric solid tumors, including brain tumors (n = 14), rhabdomyosarcomas (n = 5), neuroblastomas (n = 3), osteosarcoma (n = 1) and clear cell sarcoma of the kidney (n = 1), found WT1 and MHC class I expression in 100% and 85% of the tumor specimens, respectively [73]. This confirms that WT1 is indeed a broadly expressed tumor antigen and a suitable target for active specific immunotherapy, also in pediatric cancer patients.

All WT1 peptide vaccines listed in Table 3 were administered via the ID route and contained immunostimulatory adjuvants (Montanide ISA-51 or OK-432). WT1 peptide vaccination proved safe as no severe systemic side effects were observed. Only one patient, known to have asthma, atopic dermatitis and hay fever, suffered from a grade 3 anaphylactic shock in the study by Hirabayashi et al. [73]. Almost all patients showed an increase in WT1-specific CTLs after vaccination, but no specific correlation with clinical outcome was achieved. Clinical responses to WT1 peptide vaccination were seen in some children, including a CR in a patient with rhabdomyosarcoma who had a very good partial response after prior therapy [41] and complete molecular remissions in 3 patients with hematological malignancies who were MRD-positive prior to start of vaccination [72]. This corroborates the hypothesis that peptide vaccines, and by extension all types of cancer vaccines, perform optimally in a low disease burden setting, but not in the setting of overt disease [41,72].

Apart from WT1, the group of Pollack et al. used glioma-associated peptide vaccines administrated in conjunction with Montanide and the TLR ligand poly-ICLC in patients with HGGs [70] and LGGs [71] (Table 3). Almost all patients showed T-cell immunoreactivity to at least one vaccine-targeted glioma-associated antigen. In the HGG patient group, a median OS of 13.3 months from diagnosis was observed, which is superior to the OS previously reported for these tumors [70]. The clinical results for children with LGG were non-inferior compared to those from several other recent trials for children who have progressed after current chemotherapy schedules [71].

## 3. Therapeutic Vaccination for Pediatric Cancer: The Future

Although objective clinical responses according to the RECIST criteria have been documented in cancer vaccine studies in children, the true clinical activity of cancer vaccines is not being captured by applying these conventional response assessment criteria. For example, the phenomenon of pseudoprogression, a transient increase in tumor size due to immune cell infiltration, is falsely categorized as PD when applying RECIST, whereas it is an important indicator of clinical activity. In the study by Pollack et al., patients with pseudoprogression had a better median OS (19.5 months) than those without pseudoprogression (10.9 months) [70]. Accurate identification of pseudoprogression as opposed to true tumor progression is essential to avoid premature termination of the therapy [74]. Future clinical trials should adopt immune-related response criteria to capture pseudoprogression [75] or use OS as the main endpoint to determine clinical effectiveness [8].

Based on the results of the hitherto published clinical trials, it is clear that therapeutic cancer vaccination is weakly effective and impractical in patients with high tumor burden or rapidly growing tumors, such as pediatric sarcomas or ALL, as it takes time for the immune response to develop [37]. Patients with a low residual tumor burden have clearly improved outcomes, indicating that these patients are best suited for cancer vaccination strategies [19]. Delivering therapeutic cancer vaccines as consolidation during the period of clinical remission could help overcome the immunosuppressive effects of the tumor microenvironment [37], and has already proven to be a viable strategy to prevent tumor recurrence in some patients. Based on a search of the ClinicalTrials.gov database (http://www.clinicaltrials.gov/), most upcoming cancer vaccine studies in pediatric patients are endorsing this strategy by applying this form of immunotherapy after conventional cytoreductive therapy.

In addition to tumor burden, it is also evident that the efficacy of cancer vaccines depends on the numbers and functions of immune effector cells, most notably CTLs and NK cells [7,8]. Cytotoxic therapies, such as chemotherapy and radiation, are regarded as immunosuppressive and can negatively affect these immune effector cells [7]. Nevertheless, it is becoming increasingly clear that both chemotherapy and radiation can also have immunostimulatory effects [7,76]. For example, certain lymphodepleting chemotherapies can create a favorable cytokine milieu, characterized by a surge of T cell- and NK cell-stimulatory cytokines such as IL-15 [77]. These potential immunostimulatory effects, besides cytoreduction, provide a further rationale for timing cancer vaccines in the consolidation phase post-chemotherapy and/or radiation therapy [7].

In line with this, according to ClinicalTrials.gov, cancer vaccines in children are being increasingly combined with or after conventional treatment modalities, including chemotherapy (e.g., temozolomide as in NCT03615404, NCT03396575, NCT03334305, NCT03299309 and NCT02511132) or radiation (as in NCT03615404, NCT02722512, NCT00634231). In addition, cancer vaccines can also be rationally combined with other immunotherapies, such as checkpoint inhibitors [78]. Toxicovigilance is mandatory when exploring such combinations, especially in vulnerable patient groups such as children.

## 4. Concluding Remarks 

Several clinical trials of therapeutic cancer vaccination in the pediatric population have been published. Thus far, observations of the feasibility, safety and immunological responses are promising. Although objective clinical response rates are rather low, there are indications that this type of immunotherapy can improve survival for some aggressive and resistant forms of childhood cancers. Most of the studies described here were pilot or phase 1 trials and included a diverse study population. More homogeneous and larger patient groups will therefore be needed to realize more significant clinical outcomes and to evaluate possible short-and long-term side effects. The patients for whom immunotherapy offers the most benefit seem to be those with MRD or more indolent disease. The use of adjuvants enhances the immunogenicity of cancer vaccines, leading to better clinical outcomes. Cancer vaccines built on the current clinical trials will most probably be used in the adjuvant setting, or in combination with other treatment modalities.

## Figures and Tables

**Table 1:** Published dendritic cell (DC)-based vaccination studies involving pediatric cancer patients.

Malignancy	Ph.	*N* (age)	Vaccine Type	Admin. Route	Adjuvant Treatment	Toxicity	Immune Response	Clinical Response	Reference
**Brain**									
Relapsed brain tumors	I	7/9 (9–22 y)	autologous immature DCs whole tumor RNA-pulsed	IV/ID	/	No significant toxicity	Humoral immune response (2/7); No T-cell reactivity to DCs	PR (1/7), SD (2/7)	Caruso et al., 2004 [26]
Relapsed malignant glioma	I	12 (11–78 y)	autologous mature DCs tumor lysate-pulsed	ID	/	Gr. 4 neurotoxicity (1); Gr. 2 hematotoxicity (2); other minor toxicities (2)	Positive DTH (6/8)	OS and PFS at 36 m: 17% (mPFS = 3 m; OS = 10.5 m) RD patients (6): PR (1), SD (1) CR patients (6): CCR 3y (2)	Rutkowski et al., 2004 [51]
Recurrent GBM	I/II	56 (7–77 y)	autologous mature DCs tumor lysate-pulsed	ID	/	Gr. 4 neurotoxicity (1); Gr. 2 hematotoxicity (2)	Positive DTH	OS at 12, 24 and 36m: 37.4%, 14.8% and 11.1%	De Vleeschouwer et al., 2008 [46]
Brain tumors ^#^	I	45 (children, age n.s.)	autologous mature DCs tumor lysate-pulsed	ID	IMQ	Only minor toxicities (including fatigue, headache, fever, vomitus)	ND	mPFS: 4.4 m (HGG), 4.3 m (GBM), 4.5 m (AA) mOS: 13.5 m (HGG), 12.2 m (GBM), 18.4 m (AA)	Ardon et al., 2010 [56]
Newly diagnosed or recurrent HGG	I	3/7 (1–18 y)	autologous immature DCs tumor lysate-pulsed	ID	/	Gr. 4 ↑ alkaline phosphatase (1/3)	ND	PR (1/3), SD (2/3)	Lasky et al., 2013 [28]
Newly diagnosed DIPG	I	5/9 (4–10 y)	autologous mature DCs tumor lysate-pulsed (allogeneic)	ID	/	No significant toxicity	↑ T-cell reactivity to TL (8/9) and to TL in CSF (2/9)	ND	Benitez-Ribas et al., 2018 [35]
**Solid**									
Relapsed solid tumors	I	10/15 (3–17 y)	autologous immature DCs tumor lysate-pulsed	ID	KLH	No significant toxicity	Positive DTH (3/6); ↑ T-cell reactivity (3/7)	PR (1/15), SD (5/15)	Geiger et al., 2001 [50]
Recurrent AR and ES	I	15/16 (8–30 y)	autologous mature DCs peptide-pulsed (breakpoint)	IV	IL-2	Gr. 3 toxicity attributed to IL-2 including fever (2/15), nausea/vomiting (1/15), ↑ bilirubin (2/15), hematotoxicity (4/15)	No T-cell reactivity to peptides	PD (15/15)	Dagher et al., 2002 [36]
Advanced solid extra-cranial tumors	I	20 (7–22 y)	autologous semimature DCs tumor lysate-pulsed	SC/IN	KLH	Only minor toxicities (including fever and local injection site pain)	Positive DTH (2/9 SC, 3/6 IN); ↑ T-cell reactivity (0/3 SC, 8/8 IN)	● SC treated patients (14): - CR patients (5): CCR (4), SD (1) - PR patient (1): PD (1) - PD patients (8): MR (1), SD (1) ● IN treated patients (8): - CR patients (4): CCR (3), PD (1) - PD patients (4): PD (4)	Dohnal et al., 2007 [48]
Metastatic or recurrent AR and ES	II	30/52 (1–39 y)	autologous mature DCs peptide-pulsed (breakpoint)	IV/ID	± IL-2	Gr. IV thrombocytopenia (1); grade 3 neutropenia (6); diarrhea (2); ↑ bilirubin (1), abdominal pain (1), skin rash (3)	↑ T-cell reactivity to peptide (9/23)	● OS at 60m: - Vaccinated patients: 43% - Non-vaccinated patients: 15%	Mackall et al., 2008 [37]
Relapsed/refractory solid tumors (ES, SS, NB)	ND	5 (3–11y)	autologous mature DCs tumor lysate-pulsed	SC	KLH	No significant toxicity	Positive DTH to TL (1/5); ↑ activated CD8^+^ T cells (2/5); ↑ NK cell cytotoxic activity (3/5)	RD patient (1): CR (1; ES) SD patients (2): PD (2; SS, NB) PD patients (2): PD (2; NB)	Suminoe et al., 2009 [52]
Relapsed solid tumors (OSa, NB, ES, MB)	I	15/16 (14–30.5 y)	autologous mature DCs tumor lysate-pulsed	ID	KLH and IL-2	No DC vaccine-related toxicities	↑ T-cell reactivity to TL (4/15)	No objective tumor responses	Himoudi et al., 2012 [27]
Relapsed/refractory solid tumors (NB and sarcoma)	I/II	10/15 (2.5–15 y)	autologous matured DCs peptide-pulsed (MAGE-A1, MAGE-A3 and NY-ESO-1)	IV	IMQ	Hematotoxicity attributed to DAC (5/10); urticaria multiforme attributed to DC vaccine (1/10)	↑ T-cell reactivity (6/9)	CR (1/10), SD (1/10)	Krishnadas et al., 2015 [38]
Metastatic and recurrent high-risk sarcomas	II	29/43 (6–33 y)	autologous mature DCs tumor lysate-pulsed	SC/ID	KLH and IL-7	Gr. 2 injection site reactions attributed to DC vaccine (5/29); Transaminitis (9/29), gr. 4 fever (1) and gr. 4 anaphylaxis (1) attributed to IL-7	↑ T-cell reactivity (16/26);	● OS and PFS at 60m, respectively: - All patients: 51% and 32% - ES/RMS: 63% and 40% - NDMD: 77%	Merchant et al., 2016 [47]
**Hematological**									
AML	ND	22 (3–14 y)	autologous mature DCs + CIKs	SC	IL-2	Only minor toxicities (including fever and hives) (7)	↑ CD8^+^ T cells	MRD negativity (5)	Bai et al., 2015 [53]
Relapsed ALL	I	1 (15 y)	allogeneic mature DCs peptide-pulsed (WT1)	ID	OK-432	No significant toxicity	↑ T-cell reactivity	Relapse 4 (14 m after last vaccine)	Saito et al., 2015 [39]
Post-HSCT relapse of hematological malignancies (ALL, AML, HL)	I/II	5 (9–19 y)	allogeneic mature DCs peptide-pulsed (WT1) + DLI	SC/ID	KLH	No significant toxicity	Positive DTH to WT1 (2/5) ↑ T-cell reactivity to WT1 (3/5)	PD (5/5)	Shah et al., 2016 [40]

^#^, mixed group involving 33 high-grade gliomas (HGG), 5 medulloblastomas/primitive neuro-ectodermal tumors, 4 ependymomas and 3 atypical rhabdoid/teratoid tumors; AA, anaplastic astrocytoma; Admin., administration; AML, acute myeloid leukemia; ALL, acute lymphoblastic leukemia; AR, alveolar rhabdomyosarcoma; CIKs, cytokine-induced killer cells; CR, complete response; CCR, continued CR; CSF, cerebrospinal fluid; DAC, decitabine; DCs, dendritic cell; DIPG, diffuse intrinsic pontine glioma; DLI, donor lymphocyte infusions; DTH, delayed-type hypersensitivity; ES, Ewing’s sarcoma; GBM, glioblastoma multiforme; Gr., grade; HGG, high-grade glioma; HL, Hodgkin’s lymphoma ID, intradermal; IL-, interleukin; IMQ, imiquimod; IN, intranodal; IV, intravenous; KLH, keyhole limpet hemocyanin; m, months; MB, medulloblastoma; MR, mixed response; MRD, minimal residual disease; *N*, number of patients; NB, neuroblastoma; ND, no data; NDMD, newly diagnosed metastatic disease; NK, natural killer; n.s., not specified; OS, overall survival; OSa, osteosarcoma; PD, progressive disease; PFS, progression free survival; Ph., study phase; PR, partial response; RD, residual disease; RMS, relapsed metastatic sarcoma; SC, subcutaneous; SD, stable disease; SS, synovial sarcoma; TL, tumor lysate; WT1, Wilms’ tumor 1; y, years. Last Pubmed search: 1 January 2019.

**Table 2:** Published tumor cell vaccination studies involving pediatric cancer patients.

Malignancy	Ph.	*N* (age)	Vaccine Type	Admin. Route	Adjuvant Treatment	Toxicity	Immune Response	Clinical Response	Reference
**Solid**									
Relapsed stage IV NB	I	12/13 (2.9–11.9 y)	IL-2 gene-modified allogeneic tumor cells	SC	IL-2	Only minor toxicities (including gr. 1 inflam-matory response and panniculitis)	No ↑ CD4^+^ or CD8^+^ T cells No eosinophilia ↑ CTL activity (3/5)	PR (1), SD (7)	Bowman et al., 1998 [62]
Advanced NB with measurable disease	I	10 (11 m–17 y)	AAV IL-2 gene-modified autologous tumor cells	SC	AAV IL-2	Only minor toxicities (including gr. 1 inflam-matory response and panniculitis)	↑ CD3^+^CD4^+^, CD16^+^, eosinophilia; ↑ IgG antibodies (4/9); ↑ CTL activity (4/9)	CR (1) PR (1), SD (3)	Bowman et al., 1998 [63]
Advanced/refractory NB	I	21 (2–17 y)	IL-2/Lptn gene-modified allogeneic tumor cells	SC	IL-2 Lptn	Injection site reactions (20); Flu-like symptoms (myalgia and fever) (10)	↑ CD4^+^ T cells, NK cells, eosinophilia; ↑ IgG antibodies (15/17); ↑ IL-5 (9/13)	SD at 8 wk (6) PD at 6–9 m (21)	Rousseau et al., 2003 [66]
Recurrent stage IV NB	I	1/7 (6–13 y)	IL-2/Lptn gene-modified autologous tumor cells	SC	IL-2 Lptn	No significant toxicity	IFN-γ (2/6) and IL-5 (3/6) tumor-specific immune response	SD (1)	Russell et al., 2007 [65]
Stage IV NB in remission	I/II	13 (2–9 y)	AAV IL-2 gene-modified autologous tumor cells	SC	AAV IL-2	Gr. 1-2 injection site reactions attributed to DC vaccine	↑ CD3^+^CD4^+^, eosinophilia; IFN-γ (4/10) and IL-5 (11/12) tumor-specific immune response	CCR (4) mPFS: 13.7 ± 2.5 m	Russell et al., 2008 [64]
**Hematological**									
Newly diagnosed or relapsed/refractory ALL	I	2/9 (5–60 y)	Autologous CD40L cells	SC	/	No significant toxicity	↑ allogeneic and peptide-specific T cell reactivity in vitro	PD	Haining et al., 2005 [68]
High risk AML or ALL in cytologic remission	ND	10/44 (4–56 y)	Autologous IL-2/CD40L cells	SC	AAV IL-2	Abscess locally at the injection site (1/10)	↑ IgG antibodies (2/10) ↑ cytotoxic (5/8), Th1- (4/8) and Th2-cell (3/8) reactivity; ↑ IFN-γ and IL-5 secretion	CCR (8/10) RFS at 3y: 85% OS at 5y: 90%	Rousseau et al., 2006 [69]

AAV, adenovirus; Admin., administration; AML, acute myeloid leukemia; ALL, acute lymphoblastic leukemia; CCR, continued complete response; CD40L, CD40 ligand; CR, complete response; CTL, cytotoxic T-lymphocyte; gr., grade; IFN-γ, interferon gamma; IL-, interleukin; Lptn, lymphotactin; m, months; *N*, number of patients; NB, neuroblastoma; ND, no data; OS, overall survival; PD, progressive disease; PFS, progression free survival; Ph., study phase; PR, partial response; RFS, relapse-free survival metastatic sarcoma; SC, subcutaneous; SD, stable disease; y, years. Last Pubmed search: 1 January 2019.

**Table 3:** Published peptide vaccination studies involving pediatric cancer patients.

Malignancy	Ph.	*N* (age)	Vaccine Type	Admin. Route	Adjuvant Treatment	Toxicity	Immune Response	Clinical Response	Reference
**Brain**									
High risk glioma	I	26 (1–21 y)	GAA peptides	SC	Montanide poly-ICLC	Gr. 1–2 injection site reactions (26); flu-like symptoms (24); gr. 1 GI toxicity (8); gr. 1 leukopenia (4)	↑ T-cell reactivity to epitopes (10 to 13Rα2, 11 to EphA2 and 3 to survivin)	CCR (2) PR (2), MR (1) SD (19)	Pollack et al., 2014 [70]
Recurrent LGG	I	14 (1.9–19 y)	GAA peptides	SC	Montanide poly-ICLC	Gr. 1–2 injection site reactions (14), flu-like symptoms (13); gr. 1–2 GI toxicity (6); gr. 2 urticaria (1)	↑ T-cell reactivity to epitopes (3 to 13Rα2, 11 to EphA2 and 3 to survivin)	PR sustained (4), MR (1) SD (7)	Pollack et al., 2016 [71]
**Solid**									
Solid tumors (RS, OSa, LS, SS)	I/II	4/23 (7–19 y)	WT1 peptide	ID	Montanide	Injection site reactions (4)	↑ WT1-specific CTLs (3/4)	CR (1) SD (1)	Hashii et al., 2010 [41]
Relapsed/refractory solid tumors	I/II	9/26 (0–17 y)	WT1 peptide	ID	Montanide	Injection site reactions (9)	↑ WT1-specific CTL reactivity (4/4)	● Overt disease (4): - MR (1) - SD (1) ● High risk in CR (5): - CCR (4)	Sawada et al., 2016 [72]
Solid tumors	ND	18/24 (2–19 y)	WT1 peptide	ID	OK-432	Only minor toxicities (including gr. 1–2 injection site reactions and fever) except gr. 3 anaphylaxis (1/18)	WT1 EliSPOT (4/18)	ND	Hirabayashi et al., 2018 [73]
**Hematological**									
ALL	I/II	1/23 (9 y)	WT1 peptide	ID	Montanide	Injection site reactions (1)	No ↑ WT1-specific CTLs	PD	Hashii et al., 2010 [41]
High risk hematological malignancies	II	3 (1–13 y)	WT1 peptide	ID	Montanide	Injection site reactions (3)	↑ WT1-specific CTLs (3)	CR (2)	Hashii et al., 2012 [42]
Relapsed/refractory hematological malignancies	I/II	4/26 (0–17 y)	WT1 peptide	ID	Montanide	Injection site reactions (4)	↑ WT1-specific CTL reactivity (4/4)	● MRD positive (3): - CR (3) ● High risk in CR (1): - CCR (1)	Sawada et al., 2016 [72]

Admin., administration; ALL, acute lymphoblastic leukemia; CCR, continued complete response; CR, complete response; CTL, cytotoxic T-lymphocyte GAA, glioma-associated antigens; GI, gastrointestinal; gr., grade; ID, intradermal; LGG, low-grade glioma; LS, liposarcoma; MR, mixed response; MRD, minimal residual disease; N, number of patients; ND, no data; OSa, osteosarcoma; PD, progressive disease; Ph., study phase; poly-ICLC, polyinosinic-polycytidylic acid; PR, partial response; RS, rhabdomyosarcoma; SC, subcutaneous; SD, stable disease; SS, synovial sarcoma; WT1, Wilms’ tumor 1; y, years. Last Pubmed search: 1 January 2019.
